# Supplementary material for: Drug repurposing for aging research using model organisms
Source: Aging Cell. 2017 Jun 16;16(5):1006–15. doi: 10.1111/acel.12626 (PMC5595691; doi:10.1111/acel.12626)
Supplement: Supplementary file 7 — Data S1 Zip‐Archive of all report cards. [file ACEL-16-1006-s007.zip › RC_INE.pdf]

INE

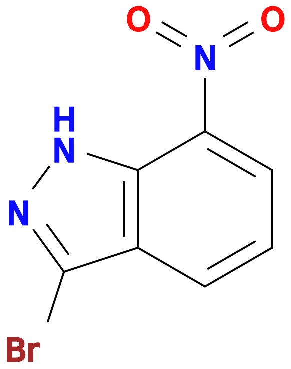

Database identifiers

ChEMBLCompound	CHEMBL479014
ZINC	ZINC03870920
eMolecules	535017

Ranking

	Rank	Score
Drosophila	411/697	0.349
C. elegans	NA	NA

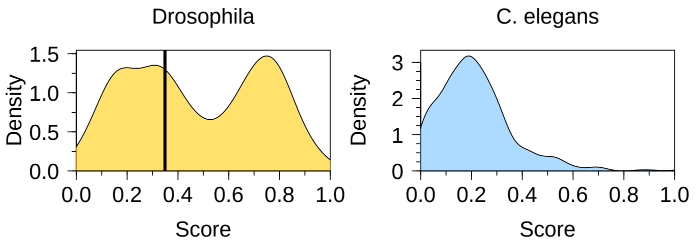

	Ageing implication	Domain conservation	Binding site conservation	Binding affinity	Bioavailability	Lipinski	Promiscuity	Purchasability	Drug approval	**Total**
Drosophila	0.624	0.958	0.947	0.492	(0.9)	0.0	-0.001	0.1	0.0	0.349
C. elegans	NA	NA	NA	NA	NA	NA	NA	NA	NA	NA

Names

- 3-bromo-7-nitroindazole
- B2050$_{s}igmaBCBcMAP01_{0}$00129
- BSPBio$_0$01502$BSPBio_0$02434
- KBioSS$_0$00222$Lopac - B - $2050
- Lopac0$_0$00161$Spectrum$1505105
- Tocris-0735

Roles

ChEBI entry None has no roles

Status

Approved drug (according to ChEMBL)	No
Number of Rule of 5 violations	0
Binding affinity to original target in log units (RF-Score prediction)	4.97
Burns *C. elegans* bioavailability prediction	-6.51

Compound Target Characteristics

Nitric oxide synthase, endothelial

Best gene implication in ageing for this target family came from gene F1LQC7 via mapping the annotation from RGD 3186 annotated in RGD 2014-03-11. Annotation GO 7568 (aging) was Inferred from Expression Pattern

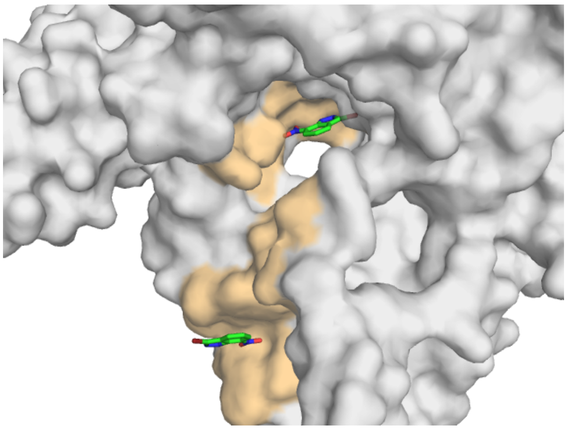

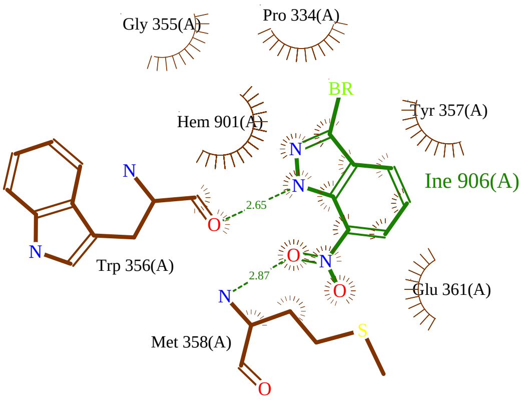

protein	amino acids contacts (binding site)
PDB:1m9r:chainA:P29474	W S V P F S G W Y M E R W A W F H Q E
sp:P29474:NOS3_HUMAN	W S V P F S G W Y M E R W A W F H Q E
tr:F1LQC7:F1LQC7_RAT	W S V P F S G W Y M E R W A W F H Q E
tr:E9Q9X4:E9Q9X4_MOUSE	W S V P F S G W Y M E R W A W F H Q E
sp:P70313:NOS3_MOUSE	W S V P F S G W Y M E R W A W F H Q E
sp:Q27571:NOS_DROME	I S M P F S G W Y M E R W I W F H Q E
sp:P16603:NCPR_YEAST	- - - - - - - - - - - - - - - - - - -

	whole protein		domain-based		contact-based	
protein	ident	simil	ident	simil	ident	simil
PDB:1m9r:chainA:P29474	1.0	1.0	1.0	1.0	1.0	1.0
sp:P29474:NOS3_HUMAN	1.0	1.0	1.0	1.0	1.0	1.0
tr:F1LQC7:F1LQC7_RAT	0.94	0.98	0.93	0.97	1.0	1.0
tr:E9Q9X4:E9Q9X4_MOUSE	0.86	0.9	0.93	0.97	1.0	1.0
sp:P70313:NOS3_MOUSE	0.94	0.98	0.93	0.97	1.0	1.0
sp:Q27571:NOS_DROME	0.4	0.69	0.49	0.78	0.84	0.91
sp:P16603:NCPR_YEAST	0.14	0.37	0.02	0.06	0.0	0.0

Nos (FBgn0011676) associated phenotypes

chemical resistant, developmental rate defective, dominant, eclosion defective, increased cell size, large body, neuroanatomy defective, partially lethal - majority die, small body, some die during pupal stage

(Information from FlyBase)

Nos (UniProt:Q27571) annotation

Function: Produces nitric oxide (NO) which is a messenger molecule with diverse functions throughout the body. Truncated isoforms (isoform 3-isoform 6) are able to form intracellular complexes with the full length protein and serve as dominant negative inhibitors of the enzyme activity.

(PubMed:11526108, PubMed:12804606, PubMed:7568075).
Cofactor: heme
Cofactor: FADNote=Binds 1 FAD. ;
Cofactor: FMNNote=Binds 1 FMN. ;
Enzyme regulation: Stimulated by calcium/calmodulin. (PubMed:7568075).
Developmental stage: Isoform 3 is expressed in larvae only. Isoform 4, isoform 5, isoform 6 and isoform 10 are expressed throughout development from embryos to adults. (PubMed:11526108).

(Information from UniProt)

Nitric oxide synthase, inducible

Best gene implication in ageing for this target family came from gene M0RDG2 via mapping the annotation from RGD 3185 annotated in RGD 2014-03-11. Annotation GO 7568 (aging) was Inferred from Expression Pattern

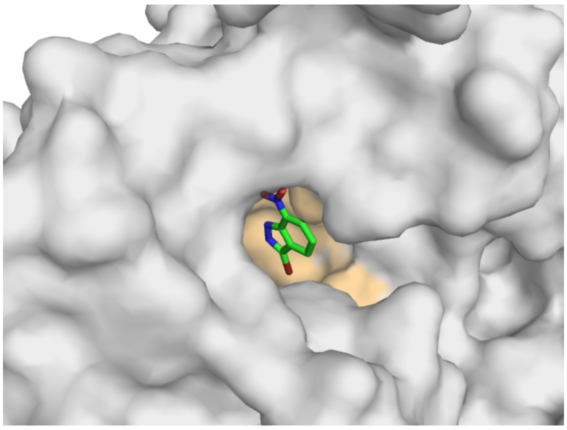

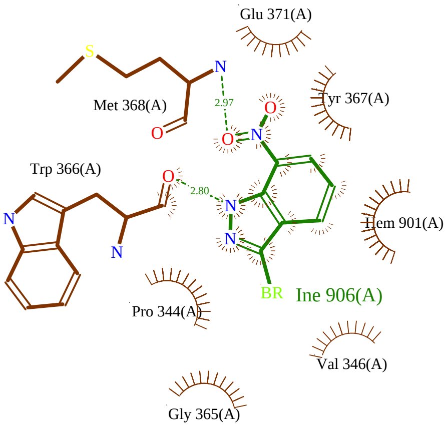

protein	amino acids contacts (binding site)
PDB:1m9t:chainA:P29477	P V F N G W Y M E
sp:P35228:NOS2_HUMAN	P V F N G W Y M E
tr:F1LSH9:F1LSH9_RAT	P V F N G W Y M E
tr:M0RDG2:M0RDG2_RAT	P V F N G W Y M E
sp:P29477:NOS2_MOUSE	P V F N G W Y M E
sp:Q27571:NOS_DROME	P V F S G W Y M E
sp:P16603:NCPR_YEAST	N I D D G D I T S

	whole protein		domain-based		contact-based	
protein	ident	simil	ident	simil	ident	simil
PDB:1m9t:chainA:P29477	1.0	1.0	1.0	1.0	1.0	1.0
sp:P35228:NOS2_HUMAN	0.8	0.93	0.92	0.98	1.0	1.0
tr:F1LSH9:F1LSH9_RAT	0.91	0.95	0.99	1.0	1.0	1.0
tr:M0RDG2:M0RDG2_RAT	0.92	0.95	0.99	1.0	1.0	1.0
sp:P29477:NOS2_MOUSE	1.0	1.0	1.0	1.0	1.0	1.0
sp:Q27571:NOS_DROME	0.38	0.68	0.62	0.91	0.89	0.95
sp:P16603:NCPR_YEAST	0.14	0.38	0.11	0.28	0.11	0.38

Nos (FBgn0011676) associated phenotypes

chemical resistant, developmental rate defective, dominant, eclosion defective, increased cell size, large body, neuroanatomy defective, partially lethal - majority die, small body, some die during pupal stage

(Information from FlyBase)

Nos (UniProt:Q27571) annotation

Function: Produces nitric oxide (NO) which is a messenger molecule with diverse functions throughout the body. Truncated isoforms (isoform 3-isoform 6) are able to form intracellular com-

plexes with the full length protein and serve as dominant negative inhibitors of the enzyme activity. (PubMed:11526108, PubMed:12804606, PubMed:7568075).
Cofactor: heme
Cofactor: FADNote=Binds 1 FAD. ;
Cofactor: FMNNote=Binds 1 FMN. ;
Enzyme regulation: Stimulated by calcium/calmodulin. (PubMed:7568075).
Developmental stage: Isoform 3 is expressed in larvae only. Isoform 4, isoform 5, isoform 6 and isoform 10 are expressed throughout development from embryos to adults. (PubMed:11526108).

(Information from UniProt)
